# Fisetin Attenuates Zinc Overload-Induced Hepatotoxicity in Mice via Autophagy-Dependent Nrf2 Activation

**DOI:** 10.3390/ijms26114978

**Published:** 2025-05-22

**Authors:** Feifei Huang, Zhonghang Wang, Mohan Zhou, Qian Zhang, Jie Feng

**Affiliations:** Key Laboratory of Nutrition and Breeding for High-Quality Animal Products of Zhejiang Province, College of Animal Science, Zhejiang University, Hangzhou 310058, China; 12217022@zju.edu.cn (F.H.); zhonghang_wang@163.com (Z.W.); chocome2@126.com (M.Z.); 12317042@zju.edu.cn (Q.Z.)

**Keywords:** Zn imbalance, liver, autophagy, Nrf2 signaling, fisetin

## Abstract

Zinc (Zn) imbalance—deficiency or overload—is implicated in hepatocyte injury, yet its mechanisms and therapeutic strategies remain incompletely understood. This study investigated Zn dyshomeostasis-induced hepatotoxicity in AML12 hepatocytes and evaluated fisetin’s protective potential in diet-induced Zn overload C57BL/6 mice for in vivo validation. In AML12 cells, both Zn deficiency and overload impaired hepatocyte viability and promoted oxidative stress, but only overload activated autophagy and the nuclear factor erythroid 2-related factor 2 (Nrf2) pathway. Fisetin, a natural flavonoid with well-documented antioxidant and anti-inflammatory properties, selectively mitigated Zn overload-induced AML12 cytotoxicity and oxidative damage by enhancing autophagic flux and Nrf2 signaling without Zn chelation, while demonstrating no effect on Zn deficiency. Specifically, fisetin required autophagy to sustain Nrf2 activation, as chloroquine abolished its protective effects. In vivo, fisetin administration (200 mg/kg BW, oral gavage) alleviated Zn overload-associated weight loss and hepatic oxidative damage in mice, paralleling its in vitro effects through reinforced autophagy–Nrf2 axis activation. The autophagy-dependent Nrf2 activation mechanism highlights fisetin’s therapeutic potential for Zn-related liver disorders.

## 1. Introduction

The liver is usually perceived as a multi-functional organ engaged primarily in bile acid biosynthesis, metabolic, nutrient storage, and detoxification activities [1,2]. The primary mediation of these functions is carried out by hepatocytes, the predominant parenchymal cells of the liver. Liver dysfunction caused by hepatocyte injury (e.g., necrosis, apoptosis, or oxidative damage) often leads to life-threatening complications. Notwithstanding the fact that the liver possesses an evolutionarily conserved and remarkable capacity for regeneration [3], it remains acutely vulnerable to damage from persistent exposure to hepatotoxic agents, including pharmaceuticals, alcohol, and environmental pollutants [4,5,6]. Consequently, hepatotoxicity continues to impose a significant global health burden [7,8], necessitating the development of novel therapeutic strategies to mitigate its progression.

Emerging evidence has highlighted the dual role of zinc (Zn), an essential trace element, in regulating hepatic physiology and pathology [9]. As a critical cofactor, Zn serves structural, catalytic, and regulatory roles in over 300 enzymes and metalloproteins [10]. Tight regulation of Zn homeostasis is indispensable for diverse physiological processes, including cellular proliferation, immune function, and metabolic regulation. Indeed, perturbations in Zn equilibrium—whether deficiency or excess—provoke hepatocyte dysfunction by exacerbating oxidative stress, impairing mitochondrial integrity, and triggering apoptotic cascades [11,12,13]. Notably, while clinical and experimental studies have established strong associations between Zn dyshomeostasis and the pathogenesis of liver injury [14,15,16], the precise molecular mechanisms underlying Zn imbalance-induced hepatotoxicity remain elusive. Moreover, therapeutic strategies to precisely correct Zn dysregulation or mitigate its downstream consequences remain underdeveloped, highlighting an urgent need for mechanistic and translational investigations.

Natural flavonoids have emerged as promising hepatoprotective candidates due to their multi-target bioactivities and favorable safety profiles [17]. Among these, fisetin—a bioactive polyphenol enriched in fruits (such as strawberries and apples) and vegetables (such as onions and cucumbers)—exhibits potent antioxidant, anti-inflammatory, and anti-apoptotic properties [18]. Functionally, fisetin requires autophagy to sustain Nrf2 activation, as chloroquine abolishes its protective effects; fisetin also activates the nuclear factor erythroid 2-related factor 2 (Nrf2) pathway to bolster cellular antioxidant defenses [19], suppresses pro-inflammatory mediators (e.g., interleukin-6, tumor necrosis factor-alpha, and monocyte chemoattractant protein-1) [20], and modulates apoptosis-related proteins such as Bcl-2 and Bcl-2-associated X protein (Bax) [21]. These pleiotropic effects have been pharmacologically validated in diverse disease models, including neurodegenerative disorders and different cancers [22,23]. Specifically for hepatic pathologies, fisetin has demonstrated efficacy in alleviating drug-induced hepatotoxicity (e.g., acetaminophen overdose) and metabolic stress in non-alcoholic fatty liver disease models [24,25]. Nevertheless, its role in mitigating Zn dyshomeostasis-driven hepatocyte injury remains unexplored.

In this study, we aimed to investigate the role of Zn imbalance in hepatic injury of mice and the potential therapeutic mechanism of fisetin. First, we utilized AML12 hepatocytes—a non-transformed cell line retaining physiologically relevant metabolic properties—to establish Zn deficiency and overload models, systematically dissecting Zn imbalance-induced hepatocyte injury. Subsequently, we comprehensively assessed fisetin’s protective effects in vitro and in vivo, with a focus on its interplay with redox homeostasis and autophagic flux.

## 2. Results

### 2.1. Establishment and Validation of Zn Imbalance Models on AML12 Cells

**Figure 1:**
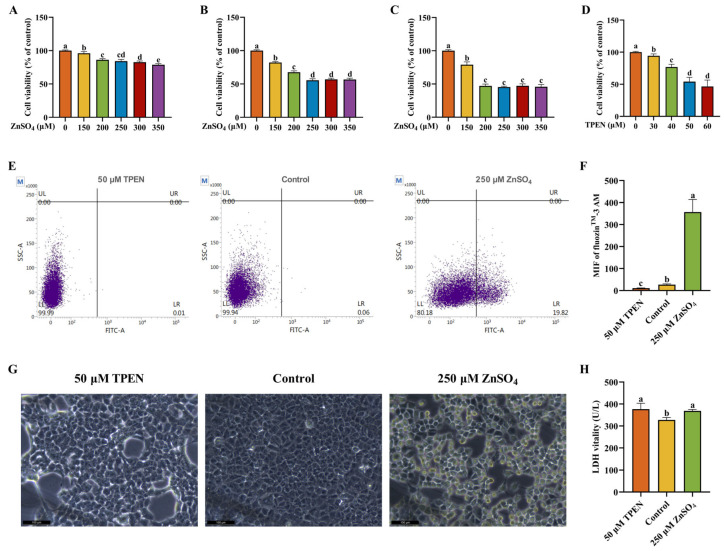
The establishment and validation of Zn imbalance models on AML12 cells. (**A**–**C**) Cell viability of various concentrations (0–350 μM) of ZnSO_4_ treatment for 2, 4 and 6 h, respectively (*n* = 6). (**D**) Cell viability of various concentrations (0–60 μM) of *N*,*N*,*N*′,*N*′-Tetrakis-(2-pyridylmethyl)-ethylenediamine (TPEN) treatment for 4 h (*n* = 6). Cell viability is expressed as a % of the control viable cell number. (**E**,**F**) Flow cytometry analysis and quantitative results of intracellular Zn^2+^ level (*n* = 3). (**G**) Morphological changes of AML12 cells. (**H**) Supernatant actate dehydrogenase (LDH) vitality of AML12 cells (*n* = 3). The results are given as the mean ± SD, and bars labeled without a common letter are significantly different.

To establish a Zn overload model, different ZnSO_4_ concentrations (150, 200, 250, 300, and 350 μM) and treatment time points (2, 4, and 6 h) were tested to verify cell viability. As shown in Figure 1A–C, exposure to varying concentrations of ZnSO_4_ for different durations consistently induced a significant reduction in cell viability. After treatment with 250 μM ZnSO_4_ for 4 h, approximately a 50% decrease in cell viability was observed relative to the control. Therefore, 250 μM ZnSO_4_ and 4 h of culture were the parameters chosen for subsequent experiments. Afterwards, on the basis of 4 h of culture, various *N*,*N*,*N*′,*N*′-Tetrakis-(2-pyridylmethyl)-ethylenediamine (TPEN) concentrations (30, 40, 50, and 60 μM) were tested to establish a Zn deficiency model. As demonstrated in Figure 1D, cell viability presented a significant reduction with the increase in TPEN concentrations. Moreover, approximately a 50% decrease in cell viability was observed relative to the control after treatment with 50 μM TPEN. Accordingly, 50 μM TPEN and 4 h of culture were the parameters chosen for subsequent experiments.

Intracellular Zn^2+^ level was detected by using a flow cytometer to verify whether the Zn imbalance model was successfully established. Compared to the control, treatment with 250 μM ZnSO_4_ significantly increased the intracellular Zn^2+^ level while 50 μM TPEN significantly decreased the intracellular Zn^2+^ level, which indicated that Zn imbalance models were effectively established (Figure 1E,F). To assess the influence of Zn imbalance on cell damage, the morphology of AML12 cells was photographed. Treatment with ZnSO_4_ and TPEN induced significant changes in morphology and cell numbers (Figure 1G). Next, to further evaluate the damage degree of AML cells, we measured the vitality of supernatant lactate dehydrogenase (LDH), which rapidly releases outside the cell when the plasma membrane is damaged and is a key feature of cellular damage. As presented in Figure 1H, compared to the control group, treatment with ZnSO_4_ and TPEN both remarkably increased the vitality of the supernatant LDH.

### 2.2. Zn Imbalance Induces Oxidative Stress in AML12 Cells

**Figure 2:**
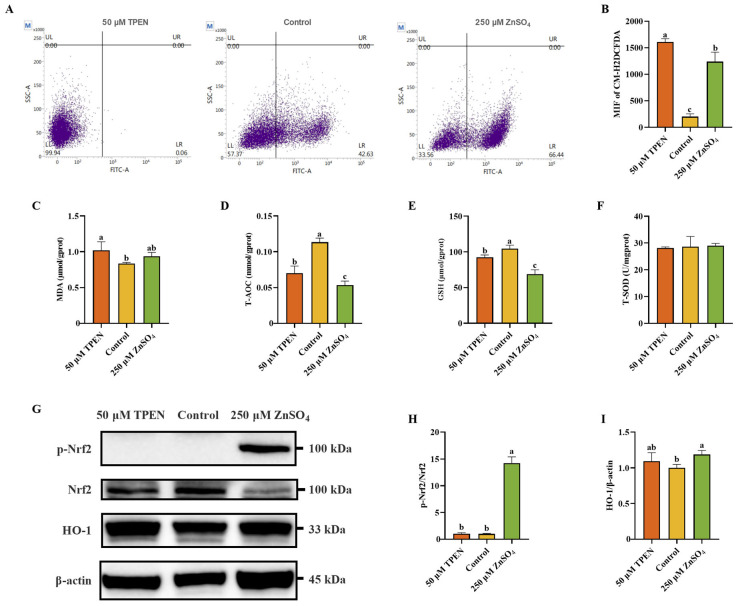
Zn imbalance induces oxidative stress in AML12 cells. (**A**,**B**) Flow cytometry analysis and quantitative results of intracellular reactive oxygen species (ROS) accumulation (*n* = 3). (**C**) Malondialdehyde (MDA) level of AML12 cells (*n* = 3). (**D**) Total antioxidant capability (T-AOC) level of AML12 cells (*n* = 3). (**E**) Glutathione (GSH) level of AML12 cells (*n* = 3). (**F**) Total superoxide dismutase (T-SOD) level of AML12 cells (*n* = 3). (**G**–**I**) Western blotting analysis and quantitative results of nuclear factor erythroid 2-related factor 2 (Nrf2) signaling pathway-related proteins Nrf2 and heme oxygenase 1 (HO-1) of AML12 cells (*n* = 3). Results are given as mean ± SD, and bars labeled without common letter are significantly different.

To delineate the impact of Zn dyshomeostasis on redox imbalance, intracellular reactive oxygen species (ROS) flux was quantitatively profiled using probe 5-(and 6)-chloromethyl-2′,7′-dichlorodihydrofluorescein diacetate (CM-H2DCFDA) fluorescence spectrometry, complemented by a systematic evaluation of antioxidant defense capacity through measurement of total antioxidant capability (T-AOC), total superoxide dismutase (T-SOD), glutathione (GSH), and malondialdehyde (MDA) using standardized commercial assays. It can be presumed from Figure 2A,B that both ZnSO_4_ and TPEN treatment drastically escalated cellular ROS accumulation compared to the control group. However, as for MDA, only TPEN treatment significantly increased the level of MDA (Figure 2C). T-AOC and GSH were remarkably decreased after the treatment of both ZnSO_4_ and TPEN, while T-SOD had no obvious exposure effect on ZnSO_4_ or TPEN (Figure 2D–F). To more precisely examine cellular oxidative stress, the expression levels of antioxidant proteins were examined. TPEN exposure had no effect on the expression of p-Nrf2 and its downstream protein heme oxygenase 1 (HO-1), while ZnSO_4_ treatment significantly enhanced the expression of p-Nrf2 and HO-1 (Figure 2G–I).

### 2.3. Effects of Zn Imbalance on Apoptosis and Autophagy in AML12 Cells

**Figure 3:**
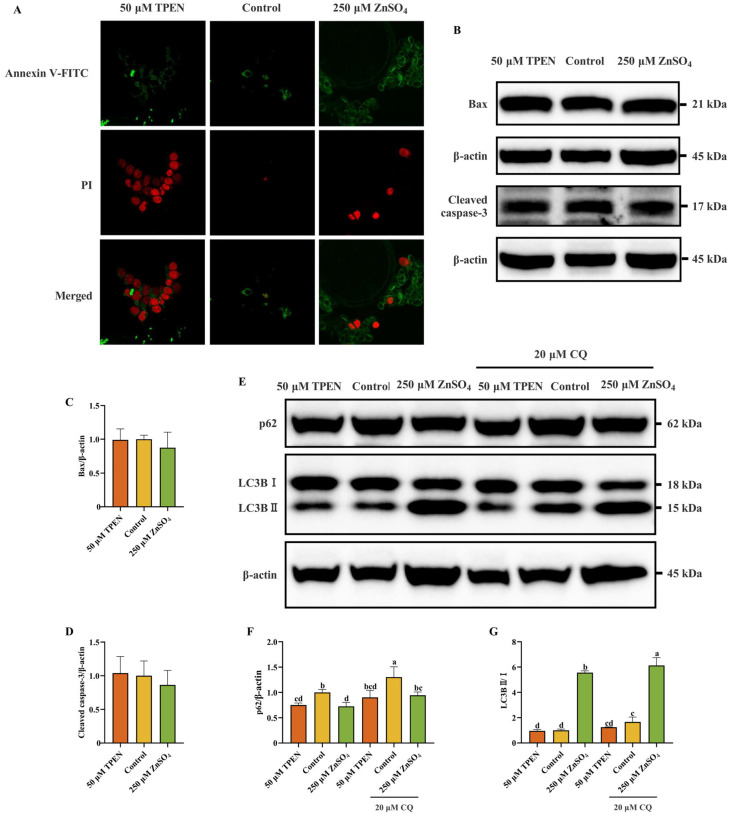
Effects of Zn imbalance on apoptosis and autophagy in AML12 cells. (**A**) Annexin V-FITC/PI staining of AML12 cells. (**B**–**D**) Western blotting analysis and quantitative results of apoptosis-associated proteins Bcl-2-associated X protein (Bax) and cleaved caspase3 of AML12 cells (*n* = 3). (**E**–**G**) Western blotting analysis and quantitative results of autophagy-associated proteins LC3B and p62 of AML12 cells (*n* = 3). Results are given as mean ± SD, and bars labeled without common letter are significantly different.

Annexin V-FITC/PI staining, the protein expression levels of Bax, cleaved caspase3, LC3B, and p62 were measured to investigate the impact of Zn imbalance on apoptosis and autophagy. As shown in Figure 3A, the percentages of apoptotic cells were slightly increased following TPEN treatment compared with the control group (Figure 3A). However, no statistically significant alterations in apoptosis-associated proteins Bax and cleaved caspase-3 expression were observed in either ZnSO_4_- or TPEN-treated groups relative to control conditions (Figure 3B–D). Furthermore, quantitative analysis of autophagic flux revealed differential responses among treatment groups. Compared to the control group, TPEN administration significantly reduced p62 protein levels without altering the LC3BII/I ratio. In contrast, ZnSO_4_ treatment not only decreased p62 expression but also increased the LC3BII/I ratio, suggesting enhanced autophagosome formation. Autophagy inhibitor chloroquine (CQ) treatment, as expected, markedly increased both p62 and the LC3BII/I ratio (Figure 3E–G). These findings collectively demonstrate that ZnSO_4_ treatment effectively enhanced cellular autophagy activity.

### 2.4. Fisetin Attenuates Zn Overload-Induced Cytotoxicity in AML12 Cells Through a Non-Chelating Way

**Figure 4:**
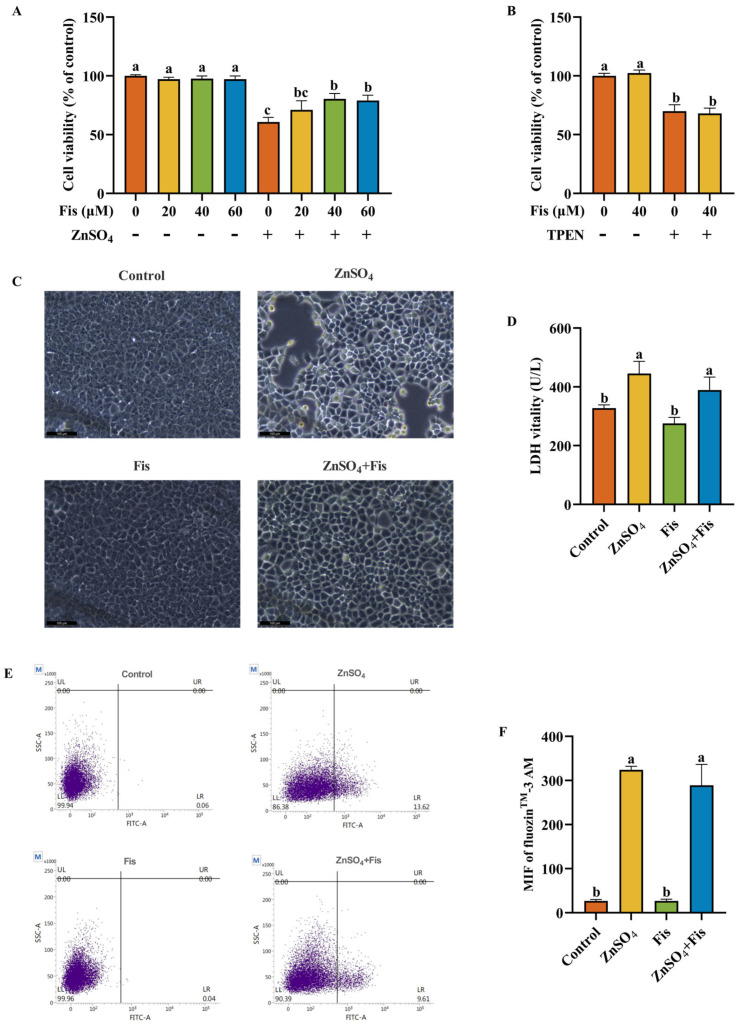
Fisetin attenuates Zn overload-induced cytotoxicity in AML12 cells through a non-chelating way. (**A**) Cell viability of various concentrations (0–60 μM) of fisetin treatment for 4 h in the presence or absence of ZnSO_4_ (*n* = 6). (**B**) Cell viability of 40 μM fisetin treatment for 4 h in the presence or absence of TPEN (*n* = 6). (**C**) Morphological changes of AML12 cells. (**D**) Supernatant LDH vitality of AML12 cells (*n* = 3). (**E**,**F**) Flow cytometry analysis and quantitative results of the intracellular Zn^2+^ level (*n* = 3). The results are given as the mean ± SD, and bars labeled without a common letter are significantly different.

To study the alleviated effect of fisetin on the decline in cell viability induced by Zn imbalance, AML12 cells were cultured with ZnSO_4_ or TPEN in the presence or absence of fisetin for 4 h before cell viability detection. In the beginning, increasing doses of fisetin (20, 40, and 60 μM) were used for co-incubation with ZnSO_4_ to test whether fisetin could minimize the negative impact on cell viability caused by Zn overload. As illustrated in Figure 4A, fisetin treatment at 40 μM demonstrated a more pronounced cytoprotective effect against ZnSO_4_-induced cellular viability inhibition compared to 20 μM, though no statistically significant difference was observed between 40 μM and 60 μM concentrations. Notably, co-treatment with all tested fisetin concentrations (20–60 μM) significantly attenuated the detrimental effects of Zn overload on cellular viability. Next, 40 μM fisetin was used to detect its function on TPEN-induced cell viability decrease. Unexpectedly, fisetin failed to recover the decline in cell viability resulting from Zn deficiency (Figure 4B). Collectively, these results uncover that fisetin had a remarkable protective effect on Zn overload-triggered cell viability decrease but did not alleviate the Zn deficiency-evoked effect on cell viability; thus, the influence of fisetin on Zn overload-induced cell injury was mainly studied in subsequent experiments. Next, morphology investigation and supernatant LDH vitality detection of AML12 cells were performed. Fisetin ameliorated significant Zn overload-induced changes in morphology and rendered the AML12 cells close to the normal morphology (Figure 4C). In addition, supernatant LDH vitality decreased after fisetin administration under conditions of ZnSO_4_ treatment (Figure 4D). Polyphenols contain abundant phenolic hydroxyl groups which could act as donors in metal–chelator interactions [26]. To rule out that the protective effects of fisetin would be due to the chelation of Zn^2+^ ions, we determined the intracellular Zn^2+^ level. Compared to the control group, ZnSO_4_ treatment remarkably increased the Zn^2+^ level. However, fisetin co-treatment exerted no influence on Zn^2+^ level when compared to the ZnSO_4_ group (Figure 4E,F). Consequently, the mitigating effect of fisetin on Zn overload was not dependent on chelated Zn^2+^ ions. These results suggest that the detrimental impact of Zn overdose on cell damage could be intensively abolished by fisetin.

### 2.5. Fisetin Attenuates Zn Overload-Induced Oxidative Stress by Activating the Nrf2 Signaling Pathway Through an Autophagy-Dependent Mechanism in AML12 Cells

**Figure 5:**
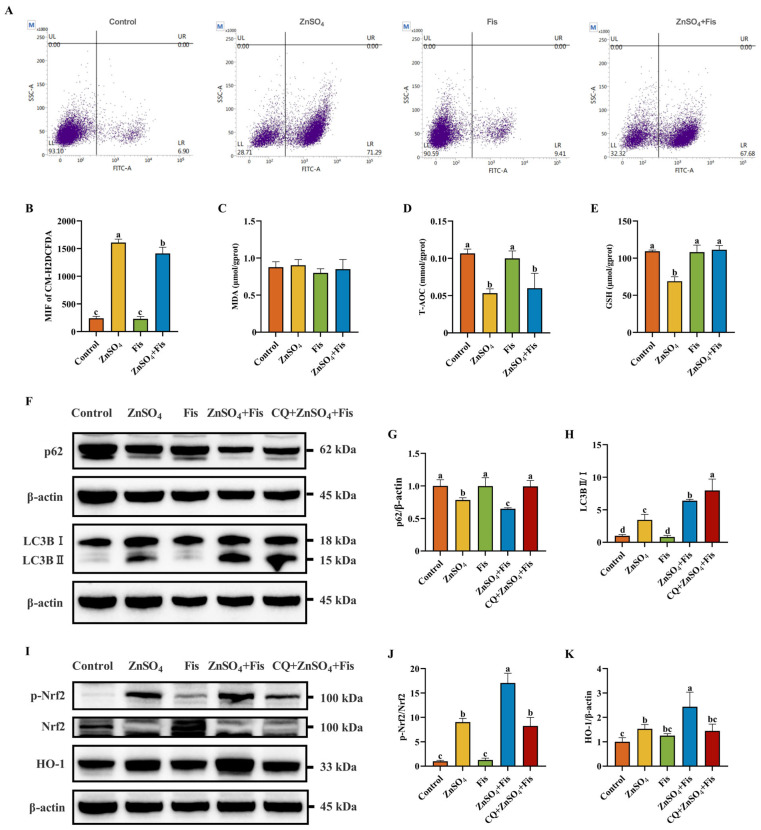
Fisetin attenuates Zn overload-induced oxidative stress by activating the Nrf2 signaling pathway through an autophagy-dependent mechanism in AML12 cells. (**A**,**B**) Flow cytometry analysis and quantitative results of intracellular ROS accumulation (*n* = 3). (**C**) MDA level of AML12 cells (*n* = 3). (**D**) T-AOC level of AML12 cells (*n* = 3). (**E**) GSH level of AML12 cells (*n* = 3). (**F**–**H**) Western blotting analysis and quantitative results of autophagy-associated proteins p62 and LC3B of AML12 cells (*n* = 4). (**I**–**K**) Western blotting analysis and quantitative results of Nrf2 signaling pathway-related proteins Nrf2 and HO-1 of AML12 cells (*n* = 4). Results are given as mean ± SD, and bars labeled without common letter are significantly different.

Fisetin has been reported to exert antioxidant effects [19], so ROS accumulation and the levels of T-AOC, GSH, and MDA were measured to reveal the response of fisetin to ZnSO_4_-induced oxidative stress. We found that ROS accumulation in ZnSO_4_-exposed AML12 cells could be mitigated by fisetin co-treatment (Figure 5A,B). In addition, we observed that fisetin co-treatment had no influence on MDA but notably reverted the ZnSO_4_-induced decline in T-AOC and GSH levels when compared to the ZnSO_4_ group (Figure 5C–E). These results suggest that fisetin is capable of attenuating Zn overload-induced ROS accumulation and antioxidant response suppression in AML12 cells.

To validate these findings and elucidate the causal relationship between autophagic flux modulation and Nrf2-mediated antioxidant response activation, we further implemented CQ and dissected Nrf2 signaling. Notably, co-treatment with fisetin augmented ZnSO_4_-induced autophagic flux, as evidenced by exacerbated p62 degradation and the amplified LC3BII/I ratio (Figure 5F–H). Consistently, fisetin further significantly enhanced ZnSO_4_-induced Nrf2 activation, while CQ co-treatment significantly attenuated p-Nrf2 levels and HO-1 expression (Figure 5I–K), despite inducing expected autophagosome accumulation (Figure 5F–H). This functional antagonism demonstrates that intact autophagic flux is a prerequisite for fisetin’s amplification of ZnSO_4_-induced Nrf2 signaling.

### 2.6. Protective Effects of Fisetin Against Zn Overload-Induced Liver Damage In Vivo

**Figure 6:**
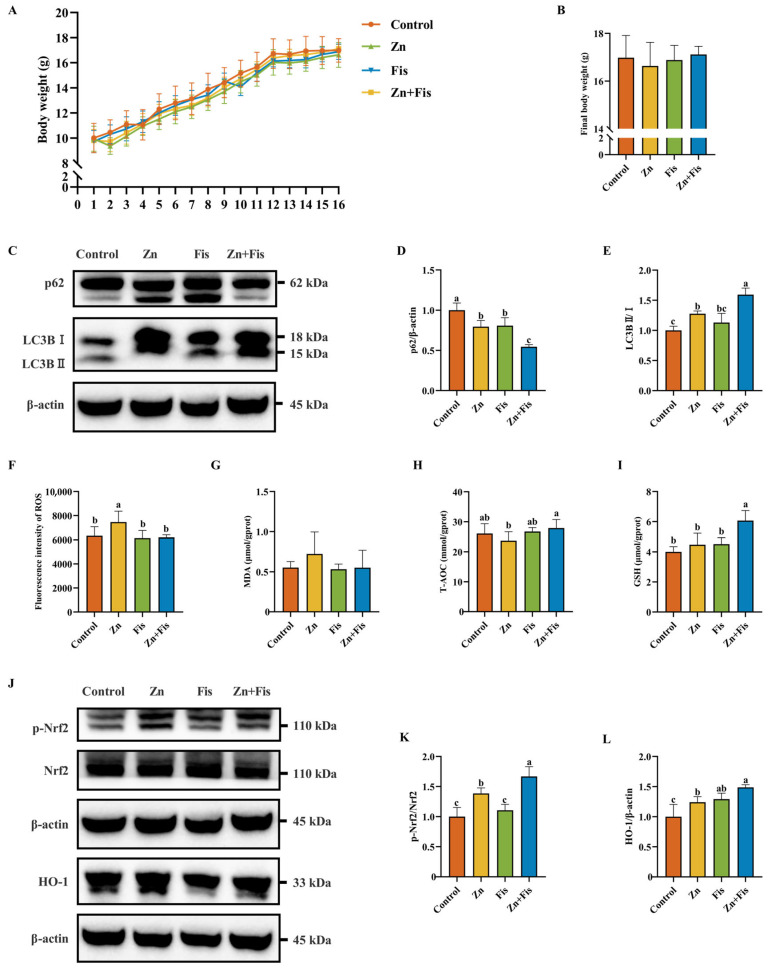
The protective effects of fisetin against Zn overload-induced liver damage in vivo. (**A**,**B**) Daily body weight and final body weight of mice throughout the entire duration of the study (*n* = 8). (**C**–**E**) Western blotting analysis and quantitative results of autophagy-associated proteins p62 and LC3B in the liver (*n* = 3). (**F**) The ROS level in the liver (*n* = 5). (**G**) The MDA level in the liver (*n* = 5). (**H**) The T-AOC level in the liver (*n* = 5). (**I**) The GSH level in the liver (*n* = 5). (**J**–**L**) Western blotting analysis and quantitative results of Nrf2 signaling pathway-related proteins Nrf2 and HO-1 in the liver (*n* = 3). The results are given as the mean ± SD, and bars labeled without a common letter are significantly different.

Compared to control mice, excess Zn exposure reduced body weight in mice, whereas fisetin supplementation partially ameliorated this weight loss, although not statistically significantly (Figure 6A,B). Consistent with in vitro findings, fisetin markedly potentiated Zn overload-induced autophagic flux, as evidenced by enhanced degradation of p62 and an elevated LC3BII/I ratio (Figure 6C–E). Oxidative stress analysis demonstrated that fisetin attenuated Zn overload-triggered ROS overproduction and MDA level changes while increasing T-AOC and GSH levels (Figure 6F–I). Moreover, fisetin potentiated the activation of the Nrf2 signaling pathway, demonstrated by enhanced p-Nrf2 and its downstream protein HO-1 (Figure 6J–L).

## 3. Discussion

The liver’s pivotal physiological roles render it a critical target organ, with hepatic pathologies posing significant threats to human health [27]. As an essential micronutrient for hepatic function, Zn homeostasis is tightly regulated by the liver, which also serves as the primary hub for Zn metabolism [28]. This study systematically elucidates the dual effects of Zn imbalance on hepatocyte viability and oxidative homeostasis, identifying fisetin as a selective therapeutic agent against Zn overload-induced hepatotoxicity.

To delineate Zn imbalance-associated hepatotoxicity, we established Zn deficiency and overload models in AML12 hepatocytes using TPEN (a Zn chelator) and ZnSO_4_, respectively. Our data reveal that both Zn deficiency and excess disrupt cellular integrity in AML12 hepatocytes, as evidenced by reduced cell viability and elevated LDH release—a hallmark of membrane destabilization. Notably, both Zn deficiency and overload induce oxidative stress, aligning with the dual role of metal ions in cellular redox regulation [29,30]. Recent studies highlight that Zn imbalance disrupts mitochondrial integrity and amplifies ROS production by interfering with antioxidant enzymes [31,32]. Similarly, in neurodegenerative diseases, Zn dyshomeostasis exacerbates lipid peroxidation and ferroptosis [33], a mechanism analogous to our findings in hepatocytes. In addition, our findings uniquely distinguish the divergent molecular mechanisms underlying Zn deficiency- versus overload-induced stress. Specifically, Zn overload robustly activated the Nrf2/HO-1 axis, a canonical antioxidant pathway, while Zn deficiency slightly enhanced apoptosis. This dichotomy suggests that Nrf2 activation serves as an adaptive mechanism uniquely mobilized during Zn overload, potentially to counteract its severe oxidative burden.

**Figure 7:**
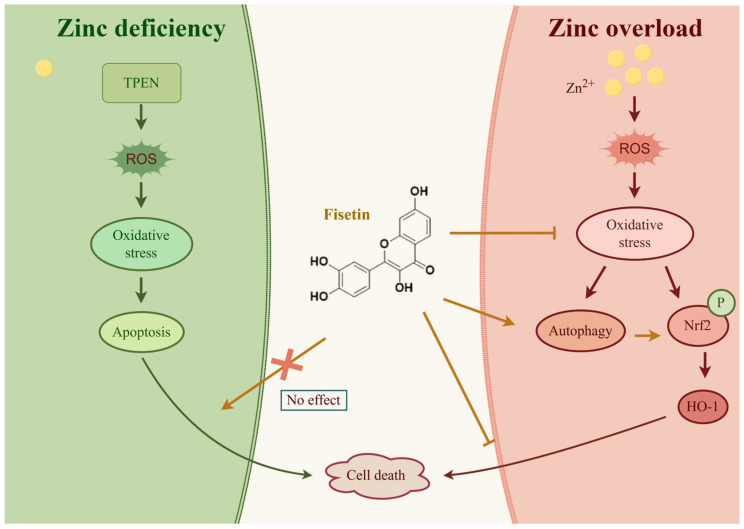
A schematic diagram of the protective mechanisms of fisetin on Zn imbalance-induced hepatotoxicity. TPEN: *N*,*N*,*N*′,*N*′-Tetrakis-(2-pyridylmethyl)-ethylenediamine; ROS: reactive oxygen species; Nrf2: nuclear factor erythroid 2-related factor 2; HO-1: heme oxygenase 1.

Intriguingly, fisetin exhibited context-dependent efficacy, reverting Zn overload-induced cytotoxicity but not mitigating Zn deficiency-related damage (Figure 7). This selectivity correlates with fisetin’s inability to compensate for the absence of Zn, a critical cofactor for numerous enzymes, while effectively amplifying endogenous antioxidant defenses under Zn overload conditions. Fisetin mediated these effects by attenuating Zn overload-driven ROS overproduction and restoring T-AOC and GSH levels, potentially through synergistic modulation of autophagy and Nrf2 signaling. Autophagy may facilitate Nrf2 activation by degrading its negative regulator Keap1 or by supplying metabolites essential for antioxidant synthesis [34,35], though further studies are needed to delineate this interplay. Similarly, quercetin activates Nrf2 via Keap1 degradation [36], while luteolin modulates autophagy [37] to alleviate liver injury. Our CQ inhibition experiments further validate that fisetin’s Nrf2 activation is autophagy-dependent, a mechanism distinct from conventional antioxidants like *N*-acetylcysteine.

The interplay between autophagy and Nrf2 in fisetin’s hepatoprotection resonates with findings in cancer and neurodegenerative models [38,39]. The present study extends this paradigm to Zn overload, proposing that fisetin-induced autophagy enables sustained Nrf2 activation. This mechanism is further supported by in vivo data showing the efficacy of fisetin in diet-induced Zn overload mice, paralleling reports where autophagy enhancers (e.g., rapamycin) improve metabolic liver damage [40,41]. Fisetin administration alleviated Zn overload-induced weight loss and hepatic oxidative damage in mice. This effect was consistent with its in vitro actions through autophagy–Nrf2 axis activation. Importantly, the efficacy of fisetin was found to be independent of Zn chelation, distinguishing it from conventional metal-chelating therapies that risk systemic Zn depletion. Instead, fisetin appears to reprogram cellular stress responses by enhancing adaptive pathways—a strategy with potential advantages in chronic Zn overload conditions such as metabolic liver diseases.

This study establishes fisetin as a selective therapeutic agent against Zn overload-induced hepatotoxicity, identifying a novel autophagy-dependent Nrf2 signaling mechanism that bridges Zn dyshomeostasis and flavonoid-based interventions. The integration of a non-transformed hepatocyte model (AML12 cells) with a diet-induced mice Zn overload model provides robust, physiologically relevant validation of pathological mechanisms and therapeutic targets, offering a foundational framework for developing targeted strategies to alleviate hepatic Zn overload injury. However, the therapeutic scope of fisetin is constrained by its inefficacy in Zn deficiency, underscoring the need for complementary approaches to address Zn-associated pathologies. Further, while autophagy is identified as critical for Nrf2 activation, the precise molecular interplay, whether through Keap1 degradation, mitophagy-mediated ROS modulation, or alternative pathways, remains unresolved. Future investigations should prioritize fisetin’s pharmacokinetic profile and long-term safety in chronic Zn overload models, as well as explore its synergy with existing chelation therapies to optimize clinical translation.

## 4. Materials and Methods

### 4.1. Cell Culture and Treatment

Mouse hepatocytes AML12 (Keygen Biotech, Nanjing, China) were grown in DMEM/F12 (Gibco, Grand Island, NY, USA) supplemented with 10% (*v*/*v*) fetal bovine serum (Gibco, Australia), 1% penicillin-streptomycin (Gibco, Grand Island, NY, USA), 0.5% ITS-G (Pricella, Wuhan, China), and 40 ng/mL dexamethasone (Sigma-Aldrich, St. Louis, MO, USA) at 37 °C in a humidified 5% CO_2_ incubator.

To establish the Zn imbalance cell model, cells were treated with Zn overload by incubating them with various concentrations (150, 200, 250, 300, and 350 μM of Zn concentration) of ZnSO_4_ (Sigma-Aldrich, St. Louis, MO, USA) for various durations (2, 4, and 6 h); cells were also treated with Zn deficiency by incubating them with various concentrations (30, 40, 50, and 60 μM) of TPEN (a chelator for Zn; MedchemExpress, Monmouth Junction, NJ, USA) for 4 h.

To investigate the effects of fisetin on liver injury induced by Zn imbalance and its mechanism, the cells were treated with ZnSO_4_ or TPEN in the presence or absence of various concentrations (20, 40, and 60 μM) of fisetin (dissolved in dimethyl sulfoxide, HY-N0182, purity 99.99%, MedchemExpress, Monmouth Junction, NJ, USA) for 4 h. Moreover, 20 μM CQ (MedchemExpress, Monmouth Junction, NJ, USA) was used for co-treatment with ZnSO_4_, TPEN, and fisetin to explore the role of autophagy in Zn imbalance-induced cellular damage.

### 4.2. Cell Viability Assay

Cell viability was determined by using the Cell Counting Kit-8 (CCK-8) assay (MedchemExpress, Monmouth Junction, NJ, USA) according to established protocols. Cells were dispensed in 96-well plates at a density of 10^4^ cells per well and incubated for 24 h. Cell-free wells and cell untreated wells were set as background correction and control groups, respectively. After the indicated treatments, 10 μL of CCK-8 reagent was added to each well and then incubated for 2 h, following which absorbance was measured at a wavelength of 450 nm using a microplate reader (Bio-Rad, Hercules, CA, USA). The data are shown as the percentage of the control.

### 4.3. Measurement of Intracellular Zn^2+^ Level

Cells were seeded in 6-well plates. After the indicated treatments, cells were lysed and centrifuged to collect the cell supernatant, then washed twice with phosphate buffer saline (PBS) before loaded with 1 μM fluozin^TM^-3, AM (Invitrogen, Carlsbad, CA, USA) for 1 h at 37 °C, and lastly washed once with PBS. The cell pellet was resuspended in 500 μL PBS and analyzed using a flow cytometer from FITC channel (FACS Calibur, BD Biosciences, San Jose, CA, USA). A minimum of 10,000 cells were analyzed per production.

### 4.4. Morphological Investigation and Quantification of Supernatant LDH Vitality

Cells were seeded in 6-well plates. After the indicated treatments, the cells were photographed immediately under a microscope (Leica, Wetzlar, Germany), and then a 100 μL supernatant sample of every well was collected and LDH was quantified using the LDH assay Kit (Nanjing Jiancheng Bioengineering Institute, Nanjing, China) according to the manufacturer’s instructions.

### 4.5. Determination of Intracellular Oxidative Stress

CM-H2DCFDA (Invitrogen, Carlsbad, CA, USA) was used to monitor the intracellular accumulation of ROS, which induces oxidative stress. Briefly, cells were seeded in 6-well plates. After the indicated treatments, cells were lysed and centrifuged to collect the cell supernatant, then washed twice with PBS before being loaded with 5 μM CM-H2DCFDA for 10 min in the dark at 37 °C, and lastly washed once with PBS. The cell pellet was resuspended in 500 μL PBS and analyzed using a flow cytometer from FITC channel (FACS Calibur, BD Biosciences, San Jose, CA, USA). A minimum of 10,000 cells were analyzed per production. The quantification of ROS in the liver was conducted in accordance with the methodology outlined by Gabbia et al. [42].

The levels of MDA and T-AOC as well as the activity of reduced GSH and T-SOD levels were measured using a specific commercial assay kit (Nanjing Jiancheng Bioengineering Institute, Nanjing, China). After treatment, the detection of cell samples was performed according to the manufacturer’s instructions.

### 4.6. Apoptosis Detection

Apoptosis was evaluated using the Annexin V-Enhanced Green Fluorescent Protein (FITC)/Propidium Iodide (PI) staining kit (Keygen Biotech, Nanjing, China). Cells (10^5^ per well) were inoculated into 12-well plates containing sterile cell climbing tablets. After the indicated treatments, the culture medium was removed and cells were washed twice with cold PBS cells. A 500 µL binding buffer was mixed with 5 µL Annexin V-FITC and 5 µL PI, and cells were stained with the mixture for 5 min at room temperature in the dark. Subsequently, the images were acquired by using a fluorescence microscope (Leica, Wetzlar, Germany). Live cells show little or no fluorescence (Annexin V^−^/PI^−^), early apoptosis cells show green fluorescence (Annexin V^+^/PI^−^), and late apoptosis cells and necrosis cells show red and green fluorescence (Annexin V^+^/PI^+^).

### 4.7. Western Blotting Analysis

Following experimental interventions, cells and liver samples were collected and lysed with RIPA lysis buffer (Beyotime Biotechnology, Shanghai, China) containing a protease inhibitor and phosphatase inhibitors. The total protein concentrations in the samples were measured by using a BCA assay kit (Keygen Biotech, Nanjing, China). Equal masses of protein samples were separated on sodium dodecyl sulfate-polyacrylamide gels and transferred from the gels onto polyvinylidene fluoride membranes. After blocking with TBST containing 5% skim milk for 1.5 h at room temperature, the membranes were incubated overnight at 4 °C with appropriate primary antibodies: β-actin (M1210-2, HUABIO, Hangzhou, China), Bax (ET1603-34, HUABIO, Hangzhou, China), cleaved caspase-3 (ET1602-47, HUABIO, Hangzhou, China), Nrf2 (16396-1-AP, Proteintech, Wuhan, China), p-Nrf2 (ab76026, Abcam, Cambridge, MA, USA), HO-1 (HA721854, HUABIO, Hangzhou, China), LC3B (db15555, Diagbio, Hangzhou, China), and p62 (HA721171, HUABIO, Hangzhou, China). On the following day, the membranes were incubated with the corresponding secondary antibody HRP goat anti-rabbit lgG (H+L) (15015, Proteintech, Wuhan, China) or HRP goat anti-mouse lgG (H+L) (15014, Proteintech, Wuhan, China) for 1.5 h at room temperature. Detection was performed by using an ECL kit (Boster, Wuhan, China) and Chemiluminescence imager (Bio-Rad, Hercules, CA, USA). Finally, the images were analyzed using Image Lab software version 6.0. The results of relative phosphorylated protein expression were calculated by normalization to total protein, while others were expressed as the abundance of each target protein relative to β-actin.

### 4.8. Animal Experiments

All of the procedures involving animals and their care have been conducted strictly according to the Institutional Animal Care and Use Committee of Zhejiang University (AP code: ZJU20240807, 16 October 2024). Three-week-old specific pathogen-free male C57BL/6 mice were purchased from Slac Laboratory Animal (Slac, Shanghai, China) and equally randomized into four groups: (1) control group (Control), standard Zn diet (30 mg Zn/kg) and daily oral administration of 0.5% carboxymethyl cellulose sodium (CMC-Na; YuanYe, Shanghai, China); (2) fisetin group (Fis), standard Zn diet and daily oral administration of fisetin (200 mg/kg BW; suspended in 0.5% CMC-Na, S31422, purity ≥ 98%, YuanYe, Shanghai, China); (3) excess Zn group (Zn), excess-Zn diet (600 mg Zn/kg) and daily oral administration of 0.5% CMC-Na; (4) excess Zn + fisetin group (Zn + Fis), excess-Zn diet and daily oral administration of fisetin. The Zn source was zinc carbonate and the dosage of Zn referred to a previously published article [43]. Liver samples were collected after 16 days and snap-frozen in liquid nitrogen for future analysis.

### 4.9. Statistical Analysis

Significant differences were analyzed by one-way ANOVA using the general linear model procedures of SPSS version 22 (SPSS Inc., Armonk, NY, USA). Data were presented as the mean ± SD. A value of *p* < 0.05 was considered statistically significant.

## 5. Conclusions

Zn overload uniquely engages autophagy and Nrf2 as compensatory responses to oxidative injury, whereas Zn deficiency primarily exacerbates redox imbalance. Fisetin selectively protects against overload-driven hepatotoxicity by amplifying autophagy-dependent Nrf2 activation, a mechanism validated in vivo. Unlike conventional chelators, fisetin modulates cellular adaptive pathways without altering Zn levels, underscoring its therapeutic specificity. These findings propose fisetin as a targeted strategy for managing Zn overload-associated hepatic pathologies.

## Data Availability

The data presented in this study are available on request from the corresponding author due to privacy.

## Abbreviations

The following abbreviations are used in this manuscript:

Bax	Bcl-2-associated X protein
CMC-Na	Carboxymethyl cellulose sodium
CM-H2DCFDA	5-(and 6)-chloromethyl-2′,7′-dichlorodihydrofluorescein diacetate
CQ	Chloroquine
GSH	Glutathione
HO-1	Heme oxygenase 1
LDH	Lactate dehydrogenase
MDA	Malondialdehyde
Nrf2	Nuclear factor erythroid 2-related factor 2
PBS	Phosphate buffer saline
ROS	Reactive oxygen species
TPEN	*N*,*N*,*N*′,*N*′-Tetrakis-(2-pyridylmethyl)-ethylenediamine
T-AOC	Total antioxidant capability
T-SOD	Total superoxide dismutase
Zn	Zinc
